# Artificial intelligence-assisted RNA-binding protein signature for prognostic stratification and therapeutic guidance in breast cancer

**DOI:** 10.3389/fimmu.2025.1583103

**Published:** 2025-04-16

**Authors:** Yunxia Zhao, Li Li, Shuqi Yuan, Zixin Meng, Jiayi Xu, Zhaogen Cai, Yijing Zhang, Xiaonan Zhang, Tao Wang

**Affiliations:** ^1^ Department of Pathophysiology, Bengbu Medical University, Longzihu, Bengbu, Anhui, China; ^2^ Department of Pathology, Bengbu Medical University, Anqing 116 Hospital, Anqing, Anhui, China; ^3^ Department of Rheumatology and Immunology, The First Affiliated Hospital of Bengbu Medical University, Bengbu, Anhui, China; ^4^ School of Clinical Medicine, Bengbu Medical University, Longzihu, Bengbu, Anhui, China; ^5^ Department of Pathology, Bengbu Medical University, Longzihu, Bengbu, Anhui, China; ^6^ Research Laboratory Center, Guizhou Provincial People’s Hospital, Nanming, Guiyang, Guizhou, China

**Keywords:** breast cancer, RNA-binding proteins, prognostic model, personalized treatment, immunotherapy

## Abstract

**Background:**

Breast cancer is the most common malignancy in women globally, with significant heterogeneity affecting prognosis and treatment. RNA-binding proteins play vital roles in tumor progression, yet their prognostic potential remains unclear. This study introduces an Artificial Intelligence-Assisted RBP Signature (AIRS) model to improve prognostic accuracy and guide personalized treatment.

**Methods:**

Data from 14 BC cohorts (9,000+ patients) were analyzed using 108 machine learning model combinations. The AIRS model, built on three key RBP genes (PGK1, MPHOSPH10, MAP2K6), stratified patients into high- and low-risk groups. Genomic alterations, single-cell transcriptomics, tumor microenvironment characteristics, and drug sensitivity were assessed to uncover AIRS-associated mechanisms.

**Results:**

The AIRS model demonstrated superior prognostic performance, surpassing 106 established signatures. High AIRS scores correlated with elevated tumor mutational burden, specific copy number alterations, and an immune-suppressive TME. Single-cell analysis revealed functional heterogeneity in epithelial cells, linking high AIRS scores to pathways like transcription factor binding. Regulatory network analysis identified key transcription factors such as MYC. Low AIRS scores predicted better responses to immune checkpoint inhibitors, while drug sensitivity analysis highlighted panobinostat and paclitaxel as potential therapies for high-risk patients.

**Conclusions:**

The AIRS model offers a robust tool for BC prognosis and treatment stratification, integrating genomic, transcriptomic, and single-cell data. It provides actionable insights for personalized therapy, paving the way for improved clinical outcomes. Future studies should validate findings across diverse populations and expand functional analyses.

## Introduction

Breast cancer (BC) is the most common malignant tumor among women worldwide. According to data released by the International Agency for Research on Cancer (IARC) of the World Health Organization, the number of new breast cancer cases globally reached 2.26 million in 2020, surpassing lung cancer to become the most prevalent cancer globally (1). The primary treatment modalities for breast cancer include surgery, radiotherapy, chemotherapy, endocrine therapy, and targeted therapy. However, the significant heterogeneity of breast cancer results in varied therapeutic responses and prognoses among patients. For breast cancer patients, prognosis evaluation is crucial for developing personalized treatment strategies. Consequently, identifying novel biomarkers and therapeutic targets is critical for advancing breast cancer diagnosis and treatment.

RNA-binding proteins (RBPs) play a pivotal role in regulating gene expression. Current research suggests that RBPs may be significantly involved in the development and progression of breast cancer (2). Although the precise mechanisms remain incompletely understood, studies have indicated that RBPs influence key processes such as cell proliferation, apoptosis, invasion, and metastasis in breast cancer (3). Additionally, RBPs may contribute to drug resistance during chemotherapy. For instance, RBPs can regulate the stability of mRNAs encoding drug target proteins. In the case of paclitaxel treatment, certain RBPs stabilize mRNAs related to tubulin, thereby reducing cancer cell sensitivity to the drug and promoting resistance (4).

To comprehensively evaluate the clinical significance of RBPs in breast cancer, this study established an Artificial Intelligence-Assisted RBP Signature (AIRS) model. Through advanced machine learning algorithms and multi-omics analysis, the study aims to elucidate the role of RBPs in breast cancer progression, identify novel biomarkers and therapeutic targets, and improve diagnostic and prognostic assessments. Furthermore, the AIRS model is designed to enhance risk stratification for breast cancer patients, providing a foundation for personalized treatment approaches.

## Methods

### Data collection and cohort selection

This research incorporated 14 breast cancer cohorts sourced from databases such as The Cancer Genome Atlas (TCGA), Gene Expression Omnibus (GEO), Metabric, and TRANSBIG. Comprehensive survival data provided by these cohorts were leveraged for in-depth analyses. RBP were obtained from the ImmReg database (5).

### Development and assessment of the RBP signature

To construct a predictive model based on RBP for breast cancer, we adopted a framework refined from earlier work, utilizing a combination of ten computational techniques (6). To construct the AIRS model, we applied 10 commonly used machine learning algorithms for survival analysis: Lasso, Ridge, Elastic Net, Stepwise Cox, Random Survival Forest (RSF), Gradient Boosting Machine (GBM), Survival Support Vector Machine (survival-SVM), CoxBoost, Supervised Principal Components (SuperPC), and Partial Least Squares Regression for Cox (plsRcox). Each algorithm was used alone or in combination with others to form 108 model configurations. These combinations were generated by pairing algorithms either sequentially or through ensemble averaging. A total of 108 configurations of these machine learning models were tested to derive an artificial intelligence-based RBP signature (AIRS). Training was conducted across multiple patient cohorts to optimize predictive accuracy using the Concordance Index (C-index). Three RBP-associated genes (PGK1, MPHOSPH10, and MAP2K6) were identified through the Random Survival Forest (RSF) algorithm and univariate Cox regression analyses. These genes formed the foundation of the AIRS, which was adjusted for enhanced prognostic prediction.

Model performance was evaluated using the Concordance Index (C-index), which measures the agreement between predicted and actual survival outcomes. The C-index was computed using the survcomp R package. Hyperparameters for each model were optimized using 10-fold cross-validation within the training cohort. The “survminer” R package’s surv_cutpoint function established the optimal cutoff for stratifying patients into high- and low-risk groups. The model’s robustness was validated across 14 independent breast cancer cohorts, representing over 9,000 patients. Additionally, the AIRS was benchmarked against 106 established breast cancer signatures, demonstrating superior prognostic performance.

### Genomic alteration analysis

Genetic variations between high- and low-risk AIRS groups were examined in the TCGA-BRCA dataset, focusing on mutation levels and Copy Number Alterations (CNA). Tumor Mutation Burden (TMB) was derived from mutation data files, and genes with mutation frequencies exceeding 5% were visualized using the maftools package. Key mutational signatures (SBS3, SBS1, SBS12, and SBS11) were emphasized. Additionally, amplified and deleted regions, including critical genes within 3q26.32 and 5q21.3, were identified.

### Single-Cell RNA sequencing analysis

Single-cell RNA sequencing (scRNA-seq) data from GEO (GSE161529) were processed using Seurat (v4.0) (7). Genes lacking expression were removed, retaining only those with detectable levels. Data normalization employed the SCTransform function, with dimensionality reduction achieved through Principal Component Analysis (PCA) and Uniform Manifold Approximation and Projection (UMAP). Clusters were identified using Seurat’s FindNeighbors and FindClusters functions, and doublets were eliminated using the DoubletFinder package (8). Cells passing quality control thresholds (e.g., mitochondrial gene content < 15%, > 500 expressed genes) were included, resulting in 51,637 cells. Cell type annotation relied on known marker genes.

### Inference of gene regulatory networks

The SCENIC approach was utilized to infer gene regulatory networks (GRNs) from scRNA-seq data. Transcription factor (TF)-target relationships were examined to define co-expression modules and identify direct targets (6). Regulatory activity scores (RAS) were computed for individual cells, and data were condensed into metacells to enhance quality and computational efficiency (9). Clustering analysis prioritized TFs with significant influence on their targets, highlighting key nodes in the GRNs.

### Tumor microenvironment and immunotherapy analysis

Tumor microenvironment (TME) differences across AIRS-defined groups were assessed using six immune infiltration algorithms implemented via the IOBR package (10). Additionally, ESTIMATE and TIDE scores provided insights into immunotherapy response potential (11, 12). Immune checkpoint levels were evaluated as predictive markers for patient response to immune checkpoint inhibitors (ICIs).

### Identification of therapeutic agents

Potential therapies for high-risk AIRS patients were identified using the Drug Repurposing Hub, where Spearman correlations between AIRS scores and gene expression were computed. CTRP and PRISM databases were utilized for drug sensitivity evaluation, while the Connectivity Map (CMap) database highlighted agents with the most therapeutic promise (CMap score < -60) (6).

### Sample collection and immunohistochemistry

Samples from 30 breast cancer patients were collected at Guizhou Provincial People’s Hospital, with tumor tissues confirmed by hematoxylin and eosin (HE) staining. Gene expression for the seven core AIRS genes was measured using qPCR to classify patients into risk groups based on the model. Immunohistochemical analysis followed established protocols, with results compared to previously published findings (13, 14).

## Results

### AI-assisted RBP signature for predictive modeling in breast cancer

**Figure 1:**
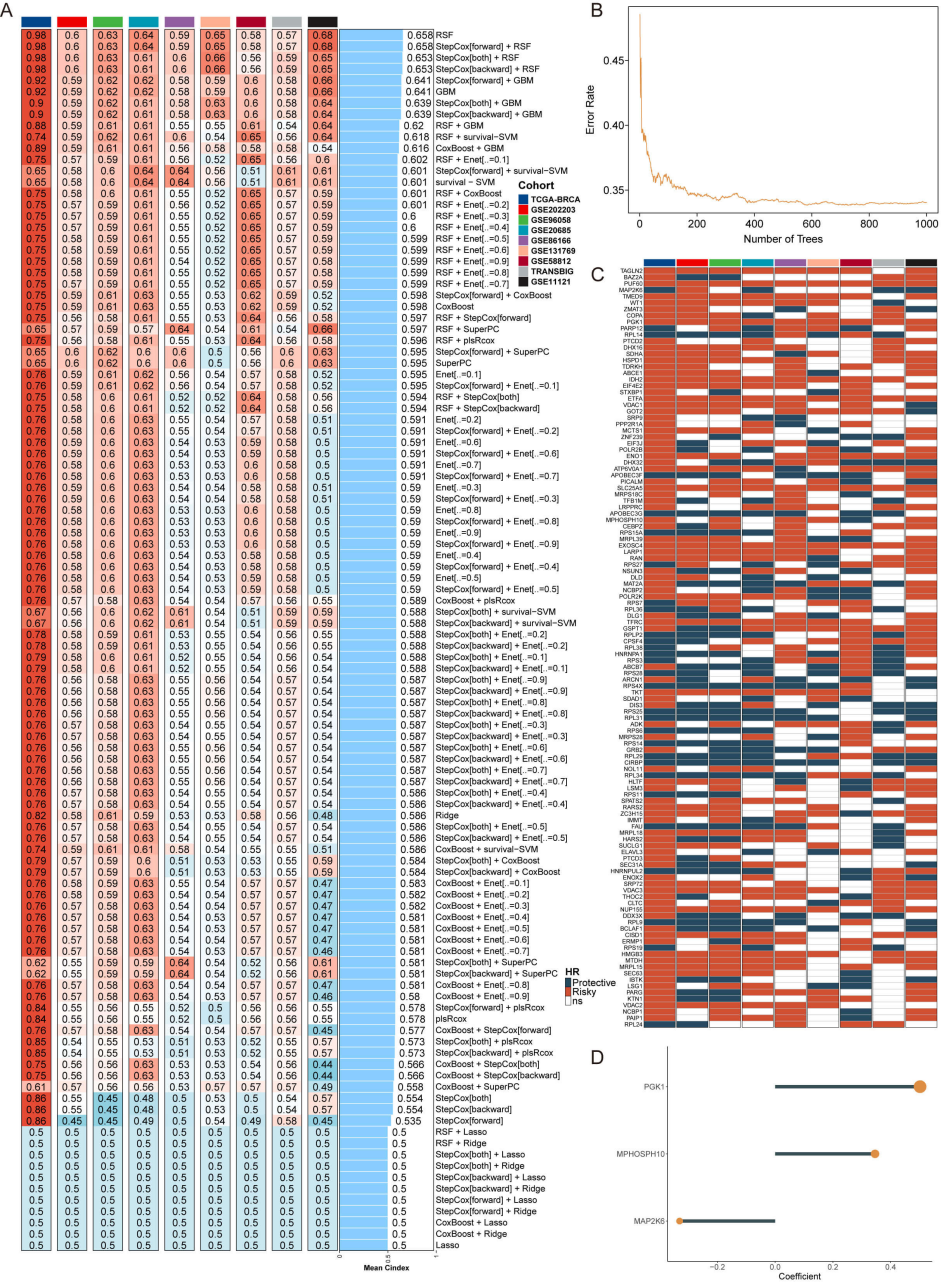
AI-Assisted RBP Signature for Predictive Modeling in Breast Cancer. **(A)** C-index values for 108 combinations of machine learning algorithms evaluated across nine cohorts. **(B)** RSF error rate calculated over 1,000 iterations. **(C)** Prognostic significance of key RBP genes across nine BC datasets. **(D)** Identification of three final RBP genes through exhaustive search, with patient risk scores computed based on gene expression levels and regression coefficients.

To comprehensively evaluate the clinical importance of RNA-binding proteins (RBPs) in breast cancer (BC), a novel artificial intelligence-based RBP signature (AIRS) was created using 108 combinations of 10 machine learning algorithms. In the TCGA-BRCA training cohort and eight validation cohorts, the average concordance index (C-index) for each algorithm combination was calculated to assess predictive accuracy (**Figure 1A**). Among these, the random survival forest (RSF) algorithm, which achieved the highest average C-index, was selected as the optimal predictive model (**Figure 1B**).

To substantiate the prognostic relevance of the identified RBP genes, univariate Cox regression analysis was conducted across all nine cohorts (**Figure 1C**). This analysis assessed the correlation between the expression of individual genes and patient survival, pinpointing key predictive factors. Subsequently, an exhaustive feature selection process evaluated all possible combinations of genes, identifying the most predictive subset. This process culminated in the selection of three pivotal RBP genes. Using the expression levels of these genes, weighted by their regression coefficients, an AIRS score was calculated for each patient (**Figure 1D**). This rigorous approach ensured the model incorporated the most significant and impactful genes, enhancing both predictive precision and clinical applicability.

Based on the AIRS model, the survminer package was utilized to classify patients into high- and low-risk groups by identifying an optimal cut-off value. Kaplan-Meier survival analysis revealed significantly higher mortality rates in the high-risk group within the TCGA-BRCA training cohort, with similar trends observed in the validation cohorts (**Supplementary Figure S1A**). The AIRS model demonstrated robust performance, achieving time-dependent area under the curve (AUC) values of 0.731, 0.715, and 0.667 at 1, 3, and 5 years, respectively, in the training cohort. Comparable results were observed across the validation cohorts, underscoring the model’s reliability and potential clinical utility (**Supplementary Figure S1B**).

### Comprehensive evaluation of AIRS predictive performance in breast cancer cohorts

**Figure 2:**
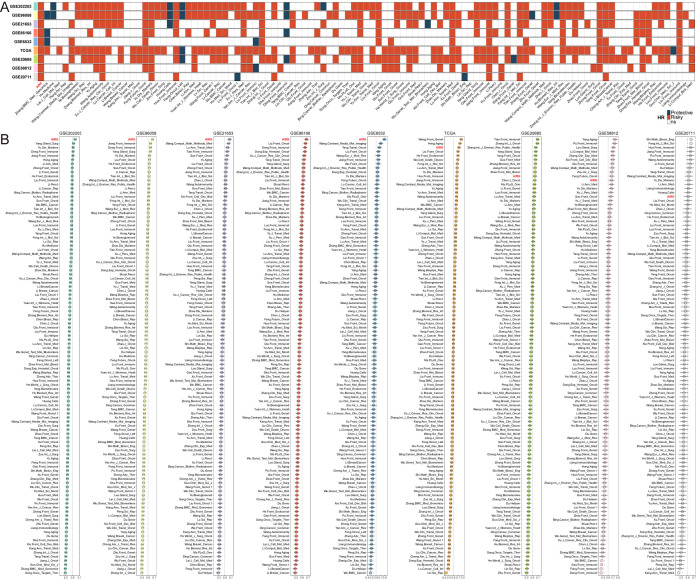
Comprehensive Evaluation of AIRS Predictive Performance in Breast Cancer Cohorts. **(A)** Stability of AIRS compared to 106 previously published models using univariate Cox regression analysis. **(B)** C-index comparisons of AIRS and 106 models across nine datasets, demonstrating AIRS’s superior predictive ability.

To further assess the predictive power of the AIRS model, its efficacy was tested against 106 published prognostic features across 10 independent breast cancer cohorts. Univariate Cox regression analysis demonstrated that AIRS was statistically significant in all cohorts, underscoring its prognostic value (**Figure 2A**). Additionally, a comparison of predictive accuracy between AIRS and the 106 features, based on the C-index across the 10 cohorts, revealed the superior performance of AIRS, highlighting its robustness and reliability in predicting breast cancer outcomes (**Figure 2B**).

Subsequently, univariate and multivariate Cox regression analyses were performed on key clinical and molecular variables, including age, menopausal status, TNM stage, pathological stage, ER, PR, HER2 expression, and the AIRS score. These analyses aimed to establish whether the prognostic value of AIRS is independent of other clinical and molecular factors. Even after adjusting for these variables, AIRS remained statistically significant for overall survival (OS), confirming its role as an independent risk factor in breast cancer prognosis (**Supplementary Figure S2A**).

A nomogram was developed by integrating AIRS, pathological stage, and age to predict survival probabilities at one, three, and five years for breast cancer patients (**Supplementary Figure S2B**). The calibration curve demonstrated excellent agreement between predicted and actual survival rates, confirming the model’s accuracy (**Supplementary Figure S2C**). Notably, the AIRS chart showed no significant difference between predicted and observed values (P > 0.05), strongly validating its predictive capability (**Supplementary Figure S2D**). Furthermore, the AIRS chart outperformed extreme prediction scenarios, demonstrating superior reliability (**Supplementary Figure S2E**). Compared with other clinicopathological factors, the AIRS model exhibited a stronger correlation with patient prognosis, emphasizing its clinical utility (**Supplementary Figure S2F**).

### Genomic alterations and prognostic implications of AIRS in breast cancer

**Figure 3:**
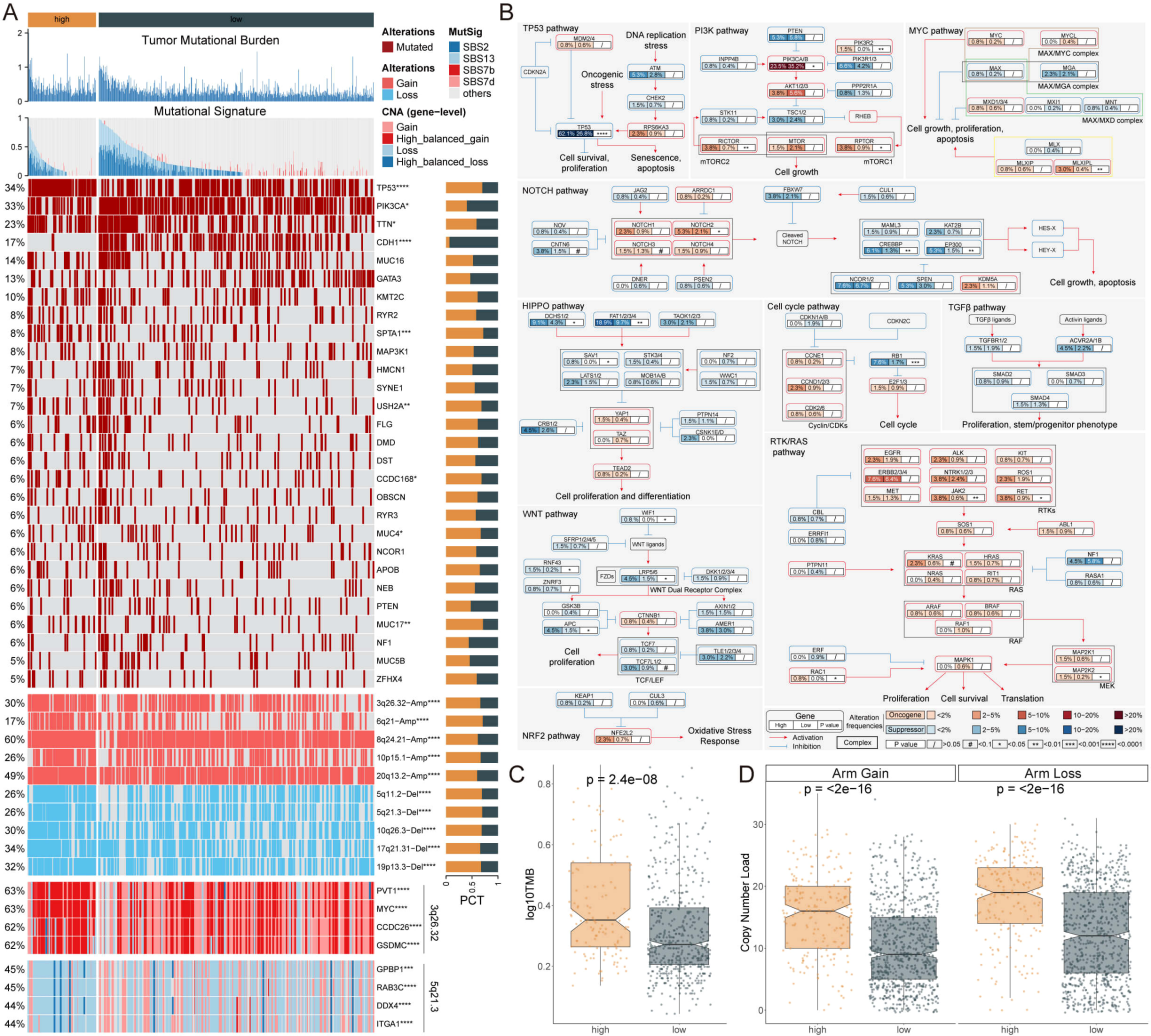
Genomic Alterations and Prognostic Implications of AIRS in Breast Cancer. **(A)** TMB, mutation characteristics, gene alterations, and copy number variations associated with AIRS. **(B)** Mutation profiles across 10 oncogenic pathways in AIRS groups. **(C)** TMB differences between high and low AIRS groups. **(D)** Copy number burden comparison between the two AIRS groups. *P < 0.05; **P < 0.01; ***P < 0.001; ****P < 0.0001.

Tumor mutational burden (TMB) analysis revealed that patients with high AIRS scores exhibited significantly elevated TMB, accompanied by diverse mutational signatures (**Figures 3A, C**). Further investigation into ten key oncogenic signaling pathways demonstrated distinct mutation patterns between high- and low-AIRS groups. Specifically, classical tumor suppressor genes, including TP53, CREBBP, FAT1/2/3/4, and RB1, were more frequently mutated in the high-AIRS group. Conversely, proto-oncogenes such as PIK3CA/B, MLXIPL, and JAK2 were predominantly mutated in the low-AIRS group (**Figures 3A, B**).

An analysis of copy number alterations (CNA) between the groups revealed a significantly higher frequency of chromosomal arm-level amplifications and deletions in patients with high AIRS scores. Amplifications were primarily observed in regions such as 3q26.32, 6q21, 8q24.21, 10p15.1, and 20q13.2, while deletions were enriched in regions including 5q11.2, 5q21.3, 10q26.3, 17q21.31, and 19p13.3 (**Figures 3A, D**).

These genomic alterations suggest that patients with high AIRS scores may have a poorer prognosis, driven by substantial gains of oncogenes such as PVT1, MYC, CCDC26, and GSDMC, coupled with concurrent losses of genes including GPBP1, RAB3C, DDX4, and ITGA1 (**Figure 3A**). These findings underscore the molecular complexity and unfavorable prognosis associated with elevated AIRS levels.

### Single-cell transcriptome analysis reveals AIRS-associated functional pathways and cellular heterogeneity

**Figure 4:**
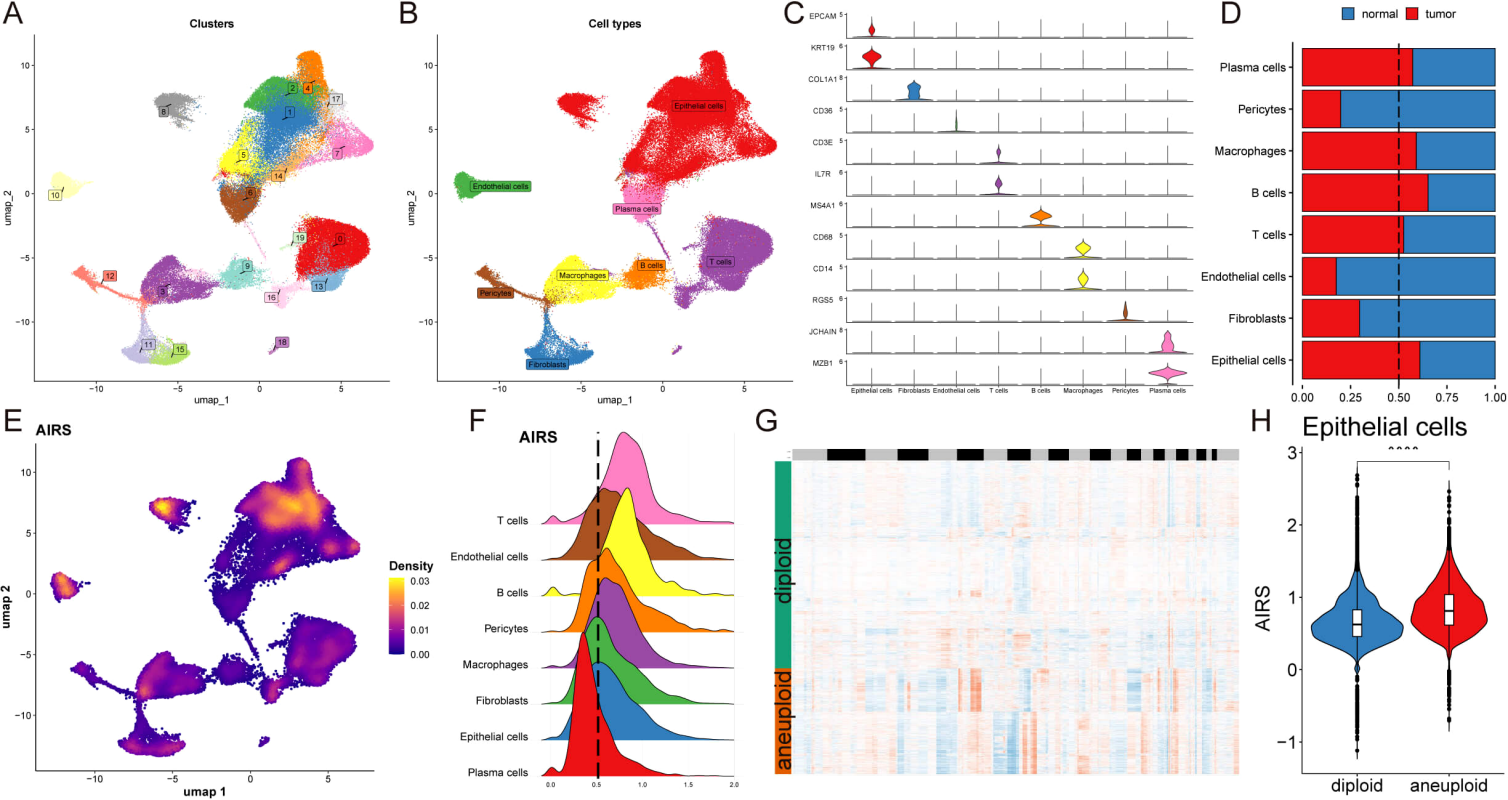
Single-Cell Transcriptome Analysis Reveals AIRS-Associated Functional Pathways and Cellular Heterogeneity. **(A)** Distribution of 20 cell clusters. **(B)** Identification of eight cell types based on marker genes. **(C)** Representative markers characterizing each cell type. **(D)** Proportions of eight cell types across tumor and normal tissues. **(E)** Distribution of AIRS scores, highlighting significant differences across cells. **(F)** Cell grouping based on epithelial cell AIRS score peaks. **(G)** CopyKat analysis comparing diploid and aneuploid cell distributions. **(H)** AIRS score comparisons between diploid and aneuploid epithelial cells.

To evaluate AIRS at the single-cell level, we performed transcriptome analysis on 12 breast cancer (BC) patient samples, encompassing both tumor and normal tissues (**Supplementary Figures S3A, B**). This analysis identified 20 distinct cell clusters classified into eight major cell types (**Figures 4A, B**). The quantity and proportion of each cell type across all patients were summarized (**Supplementary Figures S3C, D**), and representative markers for each cell type were characterized (**Figure 4C**, **Supplementary Figure S3E**). Notably, mast cells, macrophages, B cells, T cells, and epithelial cells were more abundant in tumor tissues, while other cell types were predominantly found in normal tissues (**Figure 4D**).

AIRS scores were then assigned to individual cells, revealing significant heterogeneity in their distribution (**Figure 4E**). Focusing on epithelial cells, cells were grouped based on their AIRS score peaks (**Figure 4F**). Differential gene expression analysis, combined with Gene Set Enrichment Analysis (GSEA), uncovered functional pathways associated with AIRS. In epithelial cells, which often serve as tumor progenitors, high AIRS scores were linked to pathways such as DNA-binding transcription factor binding, ubiquitin-like protein ligase binding, ribosome binding, unfolded protein binding, and protein folding chaperones. Conversely, low AIRS scores were not significantly associated with any of these pathways (**Supplementary Figure S3G**).

Further analysis using the copyKat algorithm revealed that aneuploid epithelial cells exhibited higher AIRS scores compared to diploid tumor cells, suggesting a potential link between chromosomal instability and elevated AIRS levels (**Figures 4G, H**). These findings highlight the role of AIRS in reflecting functional and genomic characteristics at the single-cell level, particularly within tumor epithelial cells.

### Construction of gene regulatory networks for AIRS using SCENIC and transcription factor analysis

**Figure 5:**
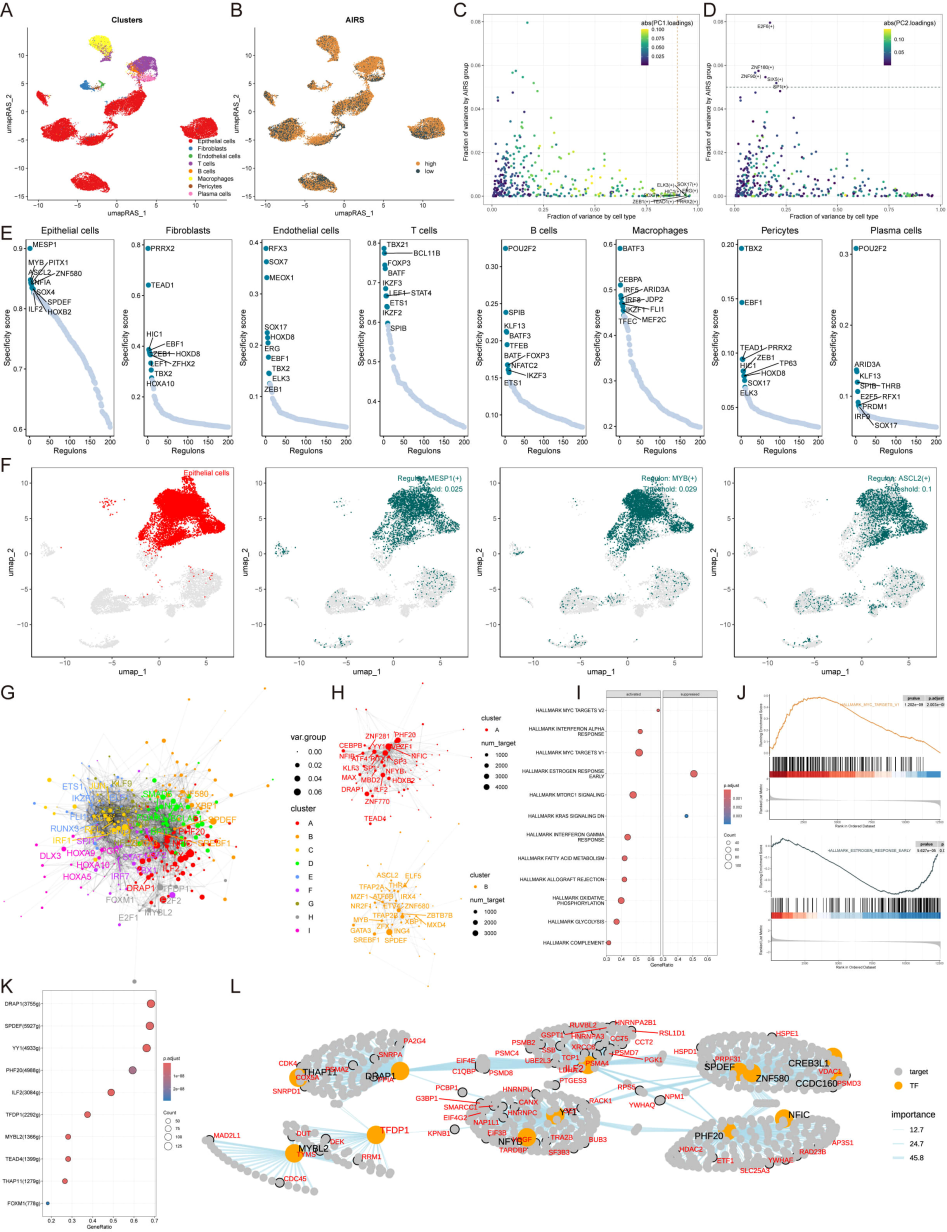
Construction of Gene Regulatory Networks for AIRS Using SCENIC and Transcription Factor Analysis. **(A)** Cell type clustering via UMAP. **(B)** SCENIC analysis transforming gene expression into transcription factor regulatory activity scores. **(C)** PCA-based decomposition identifying cell type-specific TFs (PC1). **(D)** AIRS-associated transcription factors (PC2). **(E)** Regulon specificity scores for key transcription factors across cell types. **(F)** UMAP visualization of epithelial cell-specific regulators. **(G)** Transcription factor network constructed using the Leiden algorithm. **(H)** Component-based organization of transcription factors in AIRS. **(I)** GSEA pathway analysis highlighting AIRS-related changes in epithelial cells. **(J)** Pathway-specific findings, including MYC target V1 activation and suppression of early estrogen response. **(K)** Identification of TFs associated with MYC target V1 activation. **(L)** Regulatory network diagram illustrating MYC target V1 relationships.

To establish comprehensive gene regulatory networks associated with AIRS, we applied the SCENIC pipeline, which integrates single-cell RNA-seq data with cis-regulatory sequence information. This approach transformed gene expression data into regulator activity scores (RAS) for transcription factors (TFs) (**Figures 5A, B**). Principal component analysis (PCA) with variance decomposition was then employed to uncover distinct regulatory mechanisms linked to AIRS and cellular architecture. PC1 identified TFs specific to cell types, while PC2 highlighted TFs uniquely associated with AIRS (**Figures 5C, D**). These analyses enabled us to pinpoint key regulators essential for distinguishing cell types.

Subsequently, the activity of each regulator was evaluated across different cell types, and regulator-specific scores (RSS) were calculated using Jensen-Shannon divergence (**Figure 5E**). Regulators with the highest RSS scores were selected for further functional analysis. For epithelial cells, MESP1, MYB, and ASCL2 emerged as the most specific regulators, a finding visually confirmed using UMAP plots (**Figure 5F**). Similar correlations between other cell types and their respective regulators were also identified (**Supplementary Figure S4A**).

Given the importance of transcription factor interactions in coordinating gene expression, the Leiden algorithm was employed to compare RAS scores and identify combinatorial patterns within AIRS-related TFs. This analysis grouped TFs into nine distinct components based on RAS similarity, with components A and B playing pivotal roles in AIRS regulation (**Figures 5G, H**, **Supplementary Figure S4B**). Further investigation into epithelial cell TFs driving AIRS-related transcriptional changes revealed modifications in key signaling pathways via GSEA (**Figure 5I**). For example, the MYC targets V1 pathway was activated in epithelial cells with high AIRS expression, while the estrogen response early pathway was suppressed (**Figures 5I, J**).

To elucidate the transcription factors influencing MYC targets V1, a regulatory network diagram was constructed, illustrating the interactions among these TFs and their roles in driving AIRS-related transcriptional dynamics (**Figures 5K, L**). These findings provide insights into the regulatory framework of AIRS and its impact on transcriptional landscapes within specific cell types.

### Cell-cell interaction dynamics and pathway analysis in AIRS groups

**Figure 6:**
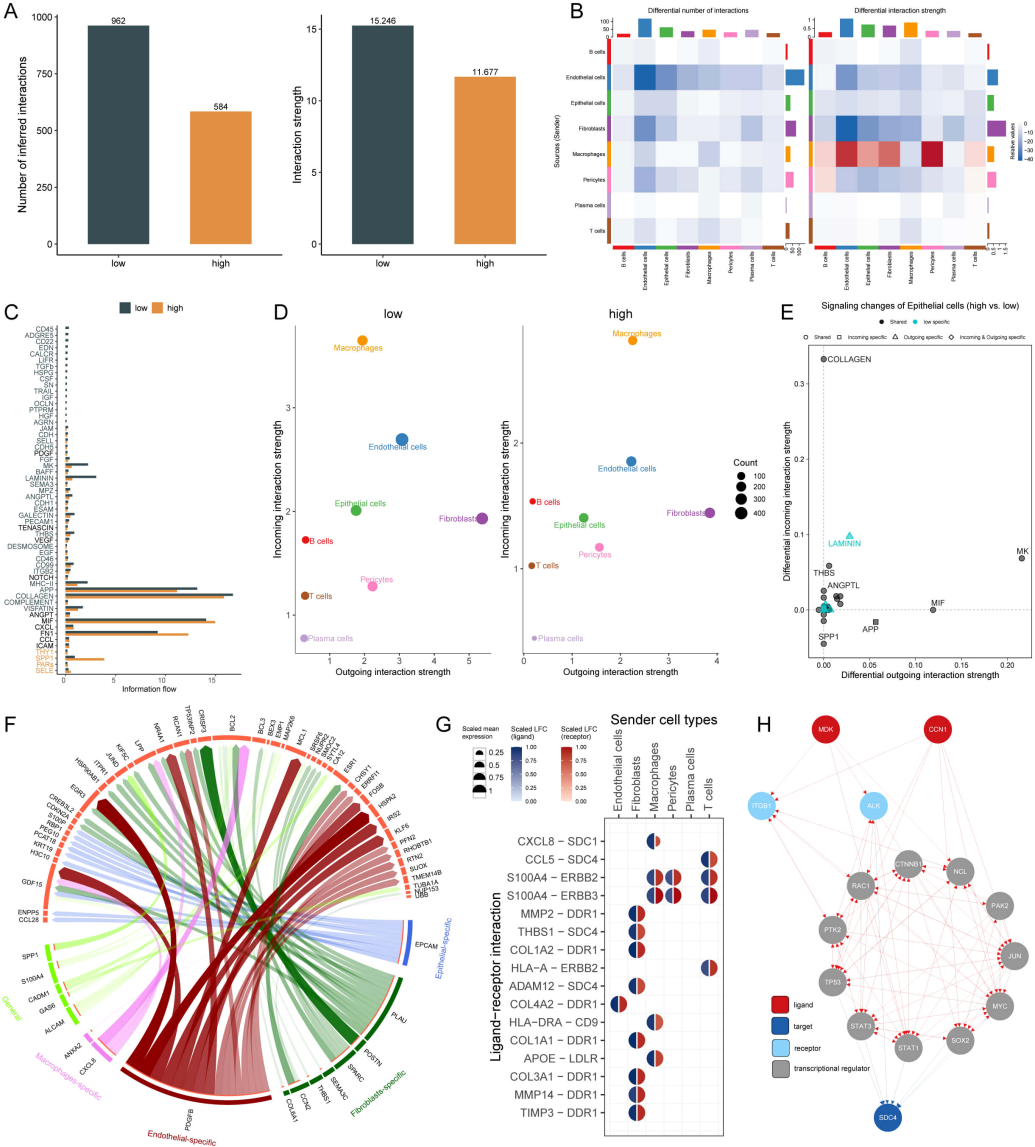
Cell-Cell Interaction Dynamics and Pathway Analysis in AIRS Groups. **(A)** Quantity and intensity of cell-cell interactions in high and low AIRS groups. **(B)** Interaction network visualization among cell types. **(C)** Differential signaling pathways between AIRS groups. **(D)** Analysis of incoming and outgoing interaction intensities among cells. **(E)** Pathways specific to AIRS in epithelial cells. **(F)** Circos diagram depicting ligand-receptor interactions. **(G)** Key ligand-receptor interactions, such as CCL8-SDC1. **(H)** Ligand network analysis showing regulatory effects on downstream transcription factors.

To investigate cell-cell communication patterns associated with AIRS, we performed CellChat analysis across eight distinct cell types. By analyzing the quantity and intensity of intercellular interactions, we observed a modest reduction in overall communication within the high AIRS group compared to the low AIRS group (**Figures 6A, B**). A comparative evaluation of 56 signaling pathways revealed differential activity: pathways such as SELE, PAPs, SPP1, and THY1 were predominantly active in the high AIRS group, whereas pathways like CD45, ADGRE5, MK, LAMININ, and CALCR were more active in the low AIRS group (**Figure 6C**).

Analysis of interaction intensity, specifically incoming and outgoing signals, revealed that epithelial cells in the high AIRS group exhibited weaker incoming interactions. This observation prompted further investigation into the relationship between specific signaling pathways in epithelial cells and AIRS. Among these, pathways such as LAMININ were identified as being closely linked to AIRS (**Figures 6D, E**).

To explore the functional dynamics of these pathways, we focused on ligand-receptor interactions and visualized key relationships using a circos diagram (**Figure 6F**). Notably, the CCL8 ligand expressed by macrophages was found to interact with the SDC1 receptor, highlighting a potentially significant regulatory axis (**Figure 6G**). Further exploration of the ligand action network demonstrated that ligands can engage in direct binding or synergistic interactions with other ligands, thereby regulating downstream transcription factors and exerting both direct and indirect effects on target pathways (**Figure 6H**).

### Immune microenvironment and immunotherapy responses in AIRS groups

**Figure 7:**
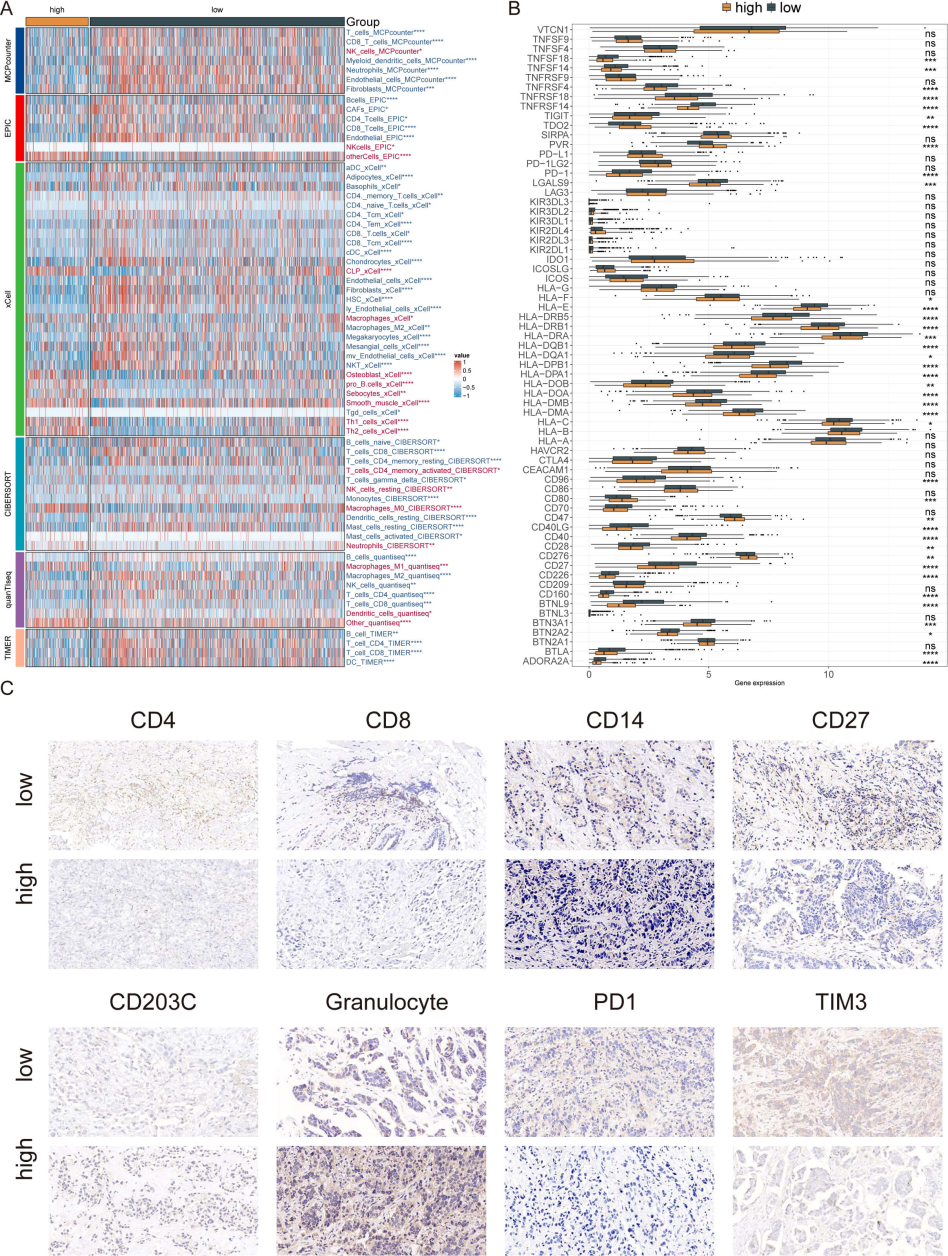
Immune Microenvironment and Immunotherapy Responses in AIRS Groups. **(A)** Heatmap showing immune cell infiltration levels in high and low AIRS groups, based on multiple algorithms. Increased infiltration is marked in red, while reduced infiltration is marked in blue. **(B)** Boxplots comparing ICI gene expression between AIRS groups (*P < 0.05; **P < 0.01; ***P < 0.001; ****P < 0.0001; ns, not significant). **(C)** Representative IHC validation of marker expression.

Given the pivotal role of the immune microenvironment in tumor progression, immune infiltration in breast cancer (BC) patients was assessed using six different algorithms. The analysis revealed reduced infiltration of CD8+ T cells and B cells in the high AIRS group (**Figure 7A**). Furthermore, the expression of immune checkpoint inhibitors (ICIs), including PD-1, TNFRSF4, CD96, and members of the HLA family, was significantly increased in the low AIRS group (**Figure 7B**). These findings were corroborated by immunohistochemistry (IHC) experiments on BC samples (**Figure 7C**).

**Figure 8:**
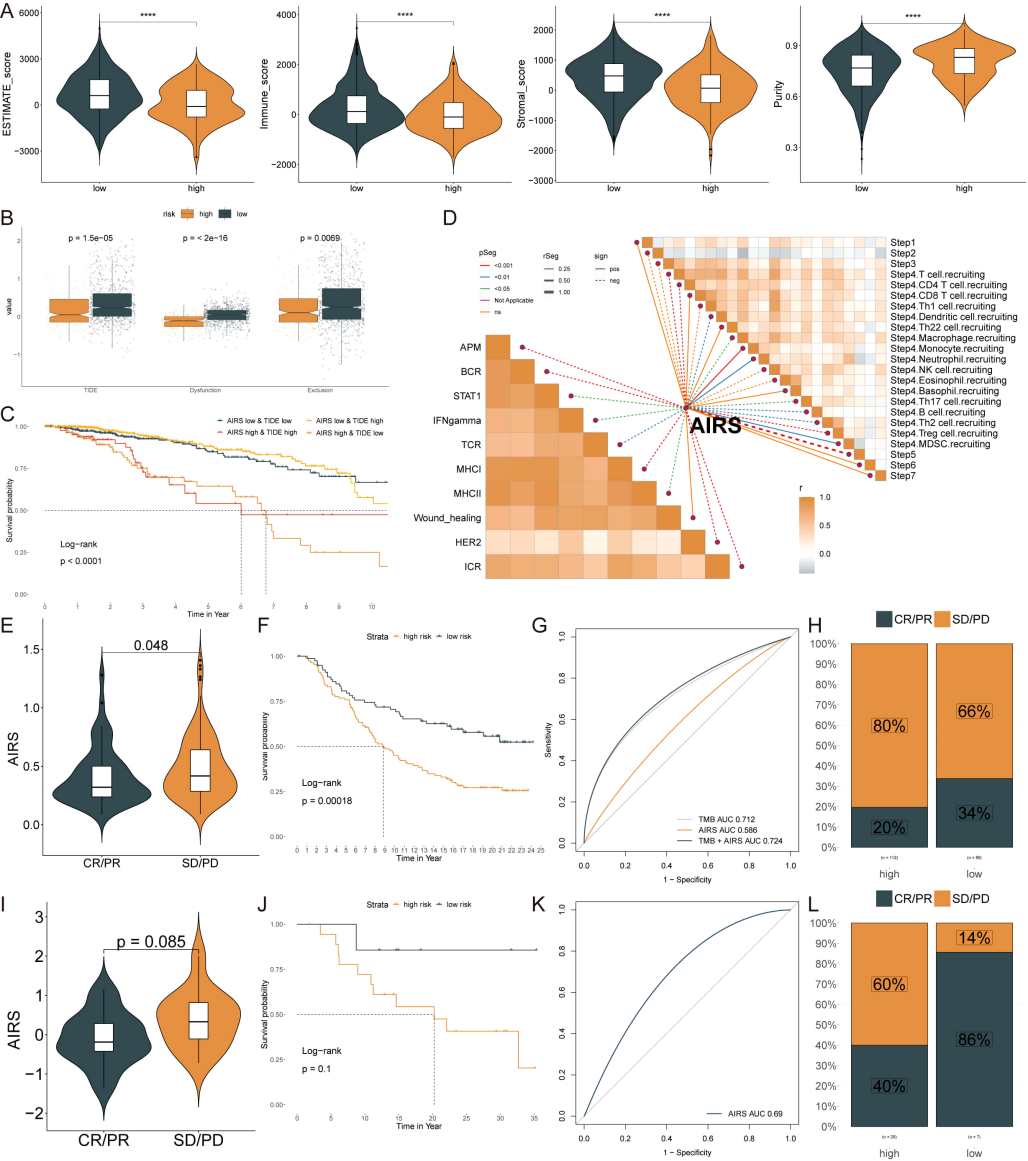
Immune Microenvironment and Immunotherapy Responses in AIRS Groups. **(A)** Immune-related scores (ESTIMATE, stromal, immune) and tumor purity across AIRS groups. **(B)** TIDE, dysfunction, and exclusion scores comparing high and low AIRS groups. **(C)** Survival probability curves based on AIRS and TIDE combinations. **(D)** Correlation analysis between AIRS and tumor immune cycle activity across ten pathways. **(E, I)** Violin plots showing AIRS levels and responses to anti-PDL1 **(E)** and anti-PD1 **(I)** therapies. **(F, J)** Survival curves for low and high AIRS patients in the anti-PDL1 **(F)** and anti-PD1 **(J)** cohorts. **(G, K)** AUC values evaluating AIRS predictive ability for TMB in anti-PDL1 **(G)** and anti-PD1 **(K)** cohorts. **(H, L)** Percentages of clinical response outcomes (CR/PR, SD/PD) in anti-PDL1 **(H)** and anti-PD1 **(L)** cohorts. ****P < 0.0001.

Using the ESTIMATE algorithm, the low AIRS group showed higher ESTIMATE, stromal, and immune scores, accompanied by lower tumor purity (p < 0.05) (**Figure 8A**). To assess immunotherapy response, various metrics such as TIDE, dysfunction, and exclusion scores were compared between the groups. Patients in the high AIRS group exhibited lower TIDE, dysfunction, and exclusion scores, suggesting reduced immunotherapy resistance (**Figure 8B**). Survival analysis showed that patients with low AIRS and high TIDE had the best prognosis (**Figure 8C**). Additionally, correlation analysis indicated higher anti-tumor immune activity in the low AIRS group compared to the high AIRS group (**Figure 8D**).

Despite the transformative impact of ICIs in cancer immunotherapy, their effectiveness in solid tumors like BC remains limited. The predictive value of AIRS levels for immune checkpoint blockade therapy was evaluated in the IMvigor210 (anti-PD-L1) and GSE78220 (anti-PD-1) cohorts. Patients with low AIRS scores demonstrated significant clinical benefits and improved survival rates when treated with anti-PD-L1 therapy (**Figures 8E–H**). Similar trends were observed for patients receiving anti-PD-1 therapy, further highlighting the predictive power of AIRS (**Figures 8I–L**).

### Identification of potential therapeutic drugs for breast cancer patients with high AIRS

**Figure 9:**
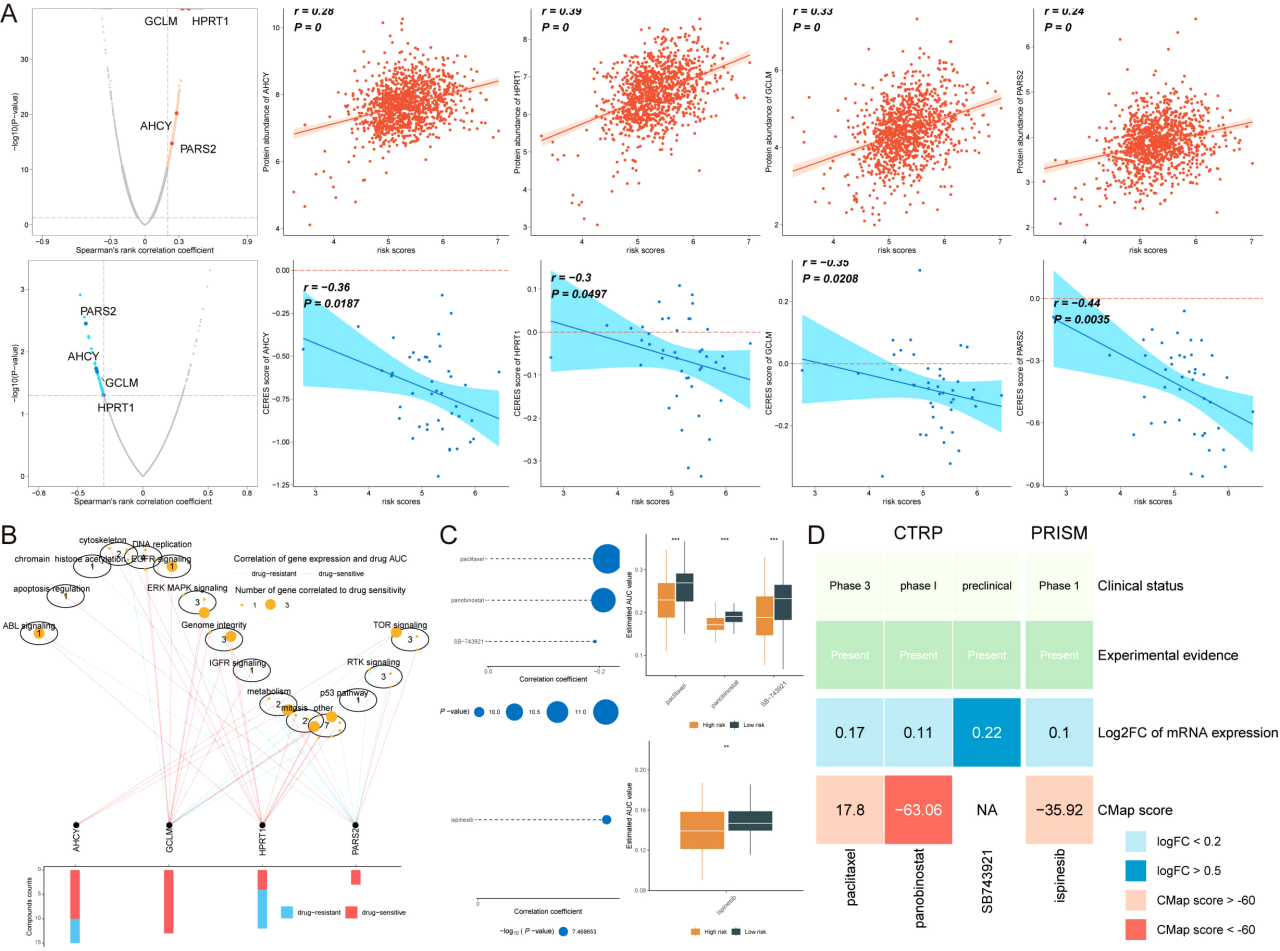
Identification of Potential Therapeutic Drugs for Breast Cancer Patients with High AIRS. **(A)** Spearman correlation shows positive (red) and negative (blue) relationships with four potential therapeutic targets. **(B)** Network analysis linking therapeutic targets to associated drug pathways. **(C)** AUC values for candidate compounds from CTRP and PRISM datasets, comparing high and low AIRS groups. **(D)** Comprehensive evaluation of clinical, experimental, and computational evidence identifying panobinostat as a key therapeutic agent for high AIRS patients. **P < 0.01.

Chemotherapy remains a cornerstone in cancer treatment. To explore potential therapeutic options for breast cancer (BC) patients with high AIRS, data from multiple datasets were analyzed. Spearman correlation analysis revealed that AIRS was positively correlated with four key targets: PARS2, AHCY, GCLM, and HPRT1, and negatively correlated with the CERES score. These findings suggest that these targets could serve as potential treatment options for patients with high AIRS (**Figure 9A**). Moreover, these targets are involved in several critical drug pathways, emphasizing their relevance as therapeutic targets for BC patients in this subgroup (**Figure 9B**).

Four candidate compounds, paclitaxel, panobinostat, SB743921, and ispinesib, were identified from the CTPR and PRISM datasets. A comparison of the area under the curve (AUC) values for these compounds between the high AIRS and low AIRS groups showed significantly lower AUC values in the high AIRS group, indicating higher sensitivity to these compounds (**Figure 9C**).

To identify the most promising therapeutic option, clinical status, experimental evidence, mRNA expression levels, and CMap scores were assessed for each compound. Based on the CMap score, panobinostat emerged as the most potential therapeutic drug for patients with high AIRS (**Figure 9D**). These findings highlight the utility of AIRS in guiding chemotherapy and targeted therapy strategies for BC patients.

## Discussion

BC is the most frequently diagnosed malignant tumor among women worldwide, with its incidence showing a continuous upward trend over recent decades, posing a significant threat to women’s lives and health (15). Despite advancements in early diagnostic techniques and comprehensive treatment strategies, many patients still face challenges such as tumor recurrence, metastasis, and poor prognosis. Thus, identifying precise and effective prognostic biomarkers and gaining deeper insights into the molecular mechanisms of tumorigenesis and progression are crucial for improving clinical outcomes in breast cancer patients.

RBPs, which play essential roles in regulating gene expression, have emerged as a research focus in breast cancer. Studies have shown that RBPs are closely associated with tumorigenesis, progression, invasion, and metastasis in breast cancer (16). By binding to specific RNA sequences, RBPs influence the expression of downstream genes and regulate tumor cell behaviors such as proliferation, apoptosis, angiogenesis, and extracellular matrix remodeling (17–20). Building on these findings, this study aimed to construct a prognostic model based on RBPs and analyze its clinical significance and underlying mechanisms. This effort seeks to provide new avenues for the clinical management of breast cancer.

This study presents a novel AIRS model, developed using 108 combinations of 10 machine learning algorithms, to predict breast cancer prognosis. Among these, the RSF algorithm, with the highest average C-index, was selected as the optimal method, ensuring superior predictive accuracy. Through rigorous feature selection, three pivotal RBP genes were identified and incorporated into the AIRS score, which stratifies patients into high- and low-risk groups with strong prognostic performance validated across nine independent cohorts. While PGK1 is a well-characterized glycolytic enzyme with established links to tumor metabolism and hypoxia responses (21), MPHOSPH10 and MAP2K6 have received comparatively less attention in breast cancer. MPHOSPH10, a nucleolar protein involved in rRNA processing, has recently been implicated in cell cycle regulation and proliferation. Dysregulation of nucleolar function is increasingly recognized in tumorigenesis. MAP2K6, a key kinase in the p38 MAPK pathway, is associated with stress and inflammatory signaling and has been linked to immune evasion and tumor progression in several cancers, including breast cancer (22). These findings support their inclusion in the AIRS model and underscore their potential relevance to tumor-immune interactions.

The AIRS model outperformed 106 established prognostic features, demonstrating its robustness and reliability in predicting patient outcomes. Furthermore, it was validated as an independent prognostic factor through multivariate Cox regression analyses. A nomogram integrating AIRS with age and pathological stage accurately predicted survival probabilities at 1, 3, and 5 years, further highlighting the model’s clinical utility. These findings underscore the potential of AI-driven models to enhance the accuracy and applicability of breast cancer prognosis, providing a transformative approach to personalized patient management. The AIRS model offers significant advancements over traditional prognostic models by addressing limitations associated with clinical indicators such as TNM stage and hormone receptor status. By incorporating multi-omics data, AIRS captures molecular and genomic features that significantly improve predictive precision.

The identification of RBPs as core components aligns with prior studies highlighting their role in tumorigenesis, metastasis, and drug resistance. For instance, RBPs regulate critical pathways such as mRNA stability and translation, influencing cancer cell behavior. Novel insights from this study include the association of AIRS scores with specific genomic alterations, such as elevated TMB and CNAs. These findings deepen our understanding of breast cancer heterogeneity and provide evidence for AIRS as a comprehensive prognostic tool.

Single-cell transcriptome analysis revealed functional heterogeneity within epithelial cells, with high AIRS scores linked to pathways involved in transcription factor binding, protein folding, and ubiquitin-like protein ligase activity (23–26). These pathways, essential for tumor progression, highlight the potential of AIRS to capture cellular-level biological mechanisms. Regulatory network analysis using SCENIC further elucidated the role of RBPs in modulating transcriptional dynamics. Key transcription factors, such as MYC and MESP1, emerged as critical regulators in AIRS-associated pathways, influencing cellular processes such as proliferation and differentiation (27–31). The association between AIRS and MYC activation aligns with literature showing MYC’s ability to regulate immune suppression and RBP expression. For instance, MYC has been shown to upregulate PGK1 and other metabolic genes that alter immune cell function (21, 32). While direct experimental validation is pending, our SCENIC and CellChat results support MYC-centered transcriptional regulation as a potential driver of the AIRS phenotype. Future studies will focus on experimental verification through gene perturbation assays. By integrating single-cell and regulatory network analyses, this study advances our understanding of RBP-mediated mechanisms in breast cancer progression.

The AIRS model sheds light on the TME and its implications for immunotherapy. High AIRS scores were associated with reduced CD8^+^ T cell and B cell infiltration, reflecting an immune-suppressive microenvironment. Conversely, low AIRS scores were linked to higher expression of immune checkpoint inhibitors (e.g., PD-1, CD96), indicating greater sensitivity to immune checkpoint blockade therapies. Data from the IMvigor210 and GSE78220 cohorts further validated AIRS as a predictive biomarker for immunotherapy. Patients with low AIRS scores demonstrated significant clinical benefits from anti-PD-1 and anti-PD-L1 therapies, underscoring the potential of AIRS to guide personalized immunotherapy strategies. While IMvigor210 and GSE78220 cohorts are from bladder cancer and melanoma, respectively, they were used due to the scarcity of publicly available breast cancer cohorts with transcriptomic and immunotherapy response data. These datasets serve as initial proof-of-concept for the predictive potential of the AIRS model in ICI-treated patients. We acknowledge this limitation and emphasize the need for future validation in breast cancer-specific cohorts receiving immune checkpoint blockade therapy.

AIRS also provides valuable insights into therapeutic decision-making. High AIRS scores were positively correlated with key targets such as PARS2, AHCY, and GCLM, suggesting potential vulnerabilities in patients with poor prognosis. Drug sensitivity analysis identified compounds like panobinostat and paclitaxel as promising therapeutic options for high AIRS patients, with lower AUC values indicating higher sensitivity. These findings demonstrate the translational relevance of AIRS in tailoring chemotherapy and targeted therapy strategies, paving the way for more effective treatment regimens in breast cancer.

This study presents a novel and comprehensive AIRS model for breast cancer prognosis, leveraging advanced machine learning algorithms and multi-omics analyses. By systematically integrating genomic, transcriptomic, and single-cell data, AIRS not only demonstrated superior predictive performance compared to 106 existing prognostic features but also revealed previously uncharacterized molecular and cellular mechanisms underlying breast cancer progression. The model’s ability to independently predict outcomes across diverse cohorts, its identification of actionable therapeutic targets, and its potential for guiding immunotherapy underscore its translational relevance and clinical utility. However, the study has certain limitations. The training and validation cohorts were primarily derived from publicly available datasets, which may limit the model’s applicability across geographically and ethnically diverse populations. Future studies should aim to validate the model in prospective datasets representing a broader range of geographical and ethnic backgrounds. Additionally, the relatively small sample size in the single-cell analysis may not fully capture the heterogeneity and complexity of breast cancer at the cellular level. Future studies should expand the dataset to include broader patient populations and conduct in-depth functional experiments to further elucidate the molecular mechanisms of RBPs. Addressing these limitations will enhance the generalizability and robustness of the AIRS model, paving the way for its integration into clinical practice.

## Conclusion

The AIRS model represents a robust and innovative prognostic tool for breast cancer, advancing our understanding of tumor biology and offering a framework for personalized treatment strategies. By informing therapeutic decisions, including immunotherapy and targeted drug selection, AIRS has the potential to transform breast cancer management and improve patient outcomes. In clinical practice, the AIRS model holds promise for complementing existing prognostic systems such as TNM staging by adding molecular-level precision. It can aid in identifying high-risk patients who may benefit from aggressive treatment or inclusion in clinical trials. Moreover, its potential to predict immunotherapy response makes it a valuable tool for guiding treatment decisions in the era of personalized oncology.

## Data Availability

The data presented in the study are deposited in the Gene Expression Omnibus (GEO) repository and the GDC portal, accession number GSE202203, GSE96058, GSE20685, GSE86166, GSE131769, GSE58812, and GSE11121.
