# Supplementary material for: Artificial intelligence-assisted RNA-binding protein signature for prognostic stratification and therapeutic guidance in breast cancer
Source: Front Immunol. 2025 Apr 16;16:1583103. doi: 10.3389/fimmu.2025.1583103 (PMC12040944; doi:10.3389/fimmu.2025.1583103)
Supplement: Supplementary file 2 [file DataSheet2.pdf]

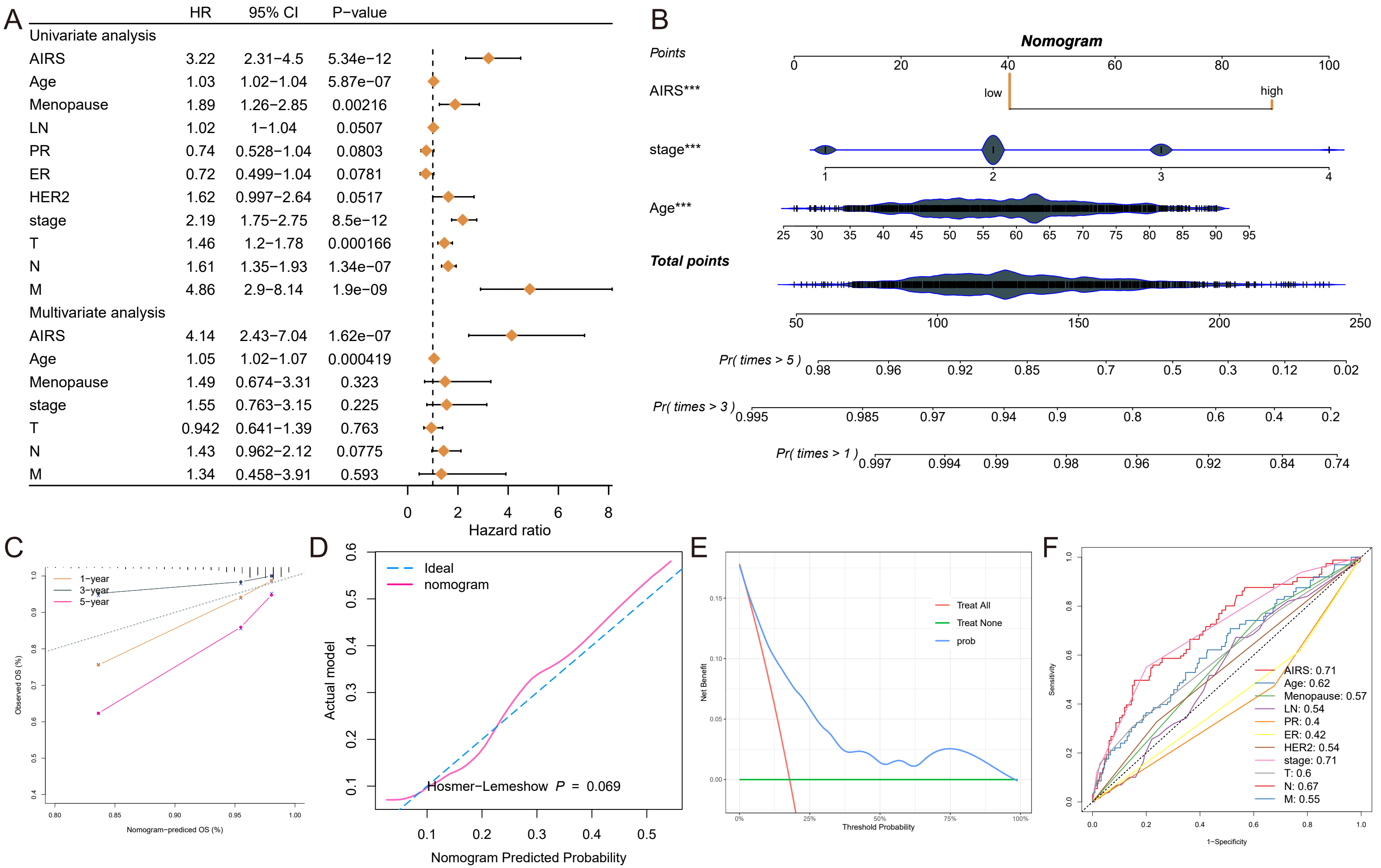

Figure S2. Prognostic features of AIRS. (A) Forest plot summarizing univariate and multivariate analyses, displaying hazard ratios (HR), 95% CI, and p-values for various prognostic factors, highlighting their statistical impact on patient outcomes. (B) Nomogram incorporating AIRS, pathological stage, and age to estimate 1-year, 3-year, and 5-year survival probabilities for breast cancer patients. (C) Calibration curve comparing the nomogram-predicted survival probabilities with observed survival rates at 1, 3, and 5 years. (D) Kernel-smoothing hazard function estimating recurrence rates in different AIRS subgroups. (E) DCA illustrating net benefits of three treatment strategies: treating all patients, treating none, or using the AIRS score to guide decisions. (F) ROC curves comparing the prognostic accuracy of the AIRS model against other clinical and pathological factors.
