# Supplementary material for: Artificial intelligence-assisted RNA-binding protein signature for prognostic stratification and therapeutic guidance in breast cancer
Source: Front Immunol. 2025 Apr 16;16:1583103. doi: 10.3389/fimmu.2025.1583103 (PMC12040944; doi:10.3389/fimmu.2025.1583103)
Supplement: Supplementary file 3 [file DataSheet3.pdf]

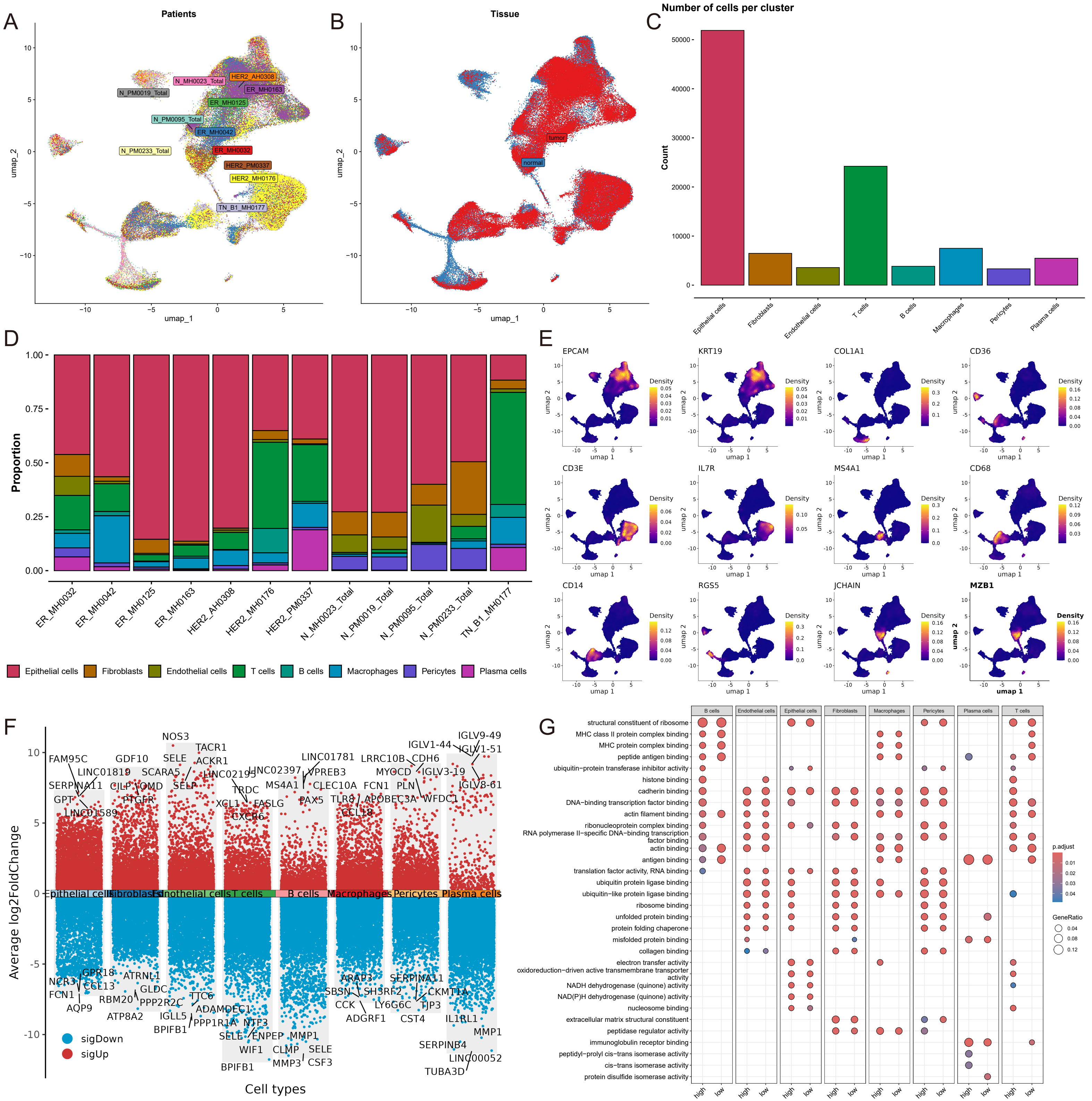

Figure S3. Comprehensive single-cell analysis atlas of breast cancer. (A) UMAP visualization displaying clustering of cells derived from 12 breast cancer patients. (B) Classification of cells based on origin, distinguishing tumor tissue (red dots) from normal tissue (blue dots). (C) Bar chart summarizing the number of cells identified in each cell cluster. (D) Bar chart showing the distribution and proportion of cell types across individual patients. (E) UMAP plots highlighting the density and expression of specific marker genes across different cell types. (F) Volcano plot visualizing differential gene expression, with significantly upregulated genes in red and downregulated genes in blue; key genes are labeled for emphasis. (G) Dot plot matrix linking differentially expressed genes in various cell types to specific biological processes.
