# Supplementary material for: Artificial intelligence-assisted RNA-binding protein signature for prognostic stratification and therapeutic guidance in breast cancer
Source: Front Immunol. 2025 Apr 16;16:1583103. doi: 10.3389/fimmu.2025.1583103 (PMC12040944; doi:10.3389/fimmu.2025.1583103)
Supplement: Supplementary file 4 [file DataSheet4.pdf]

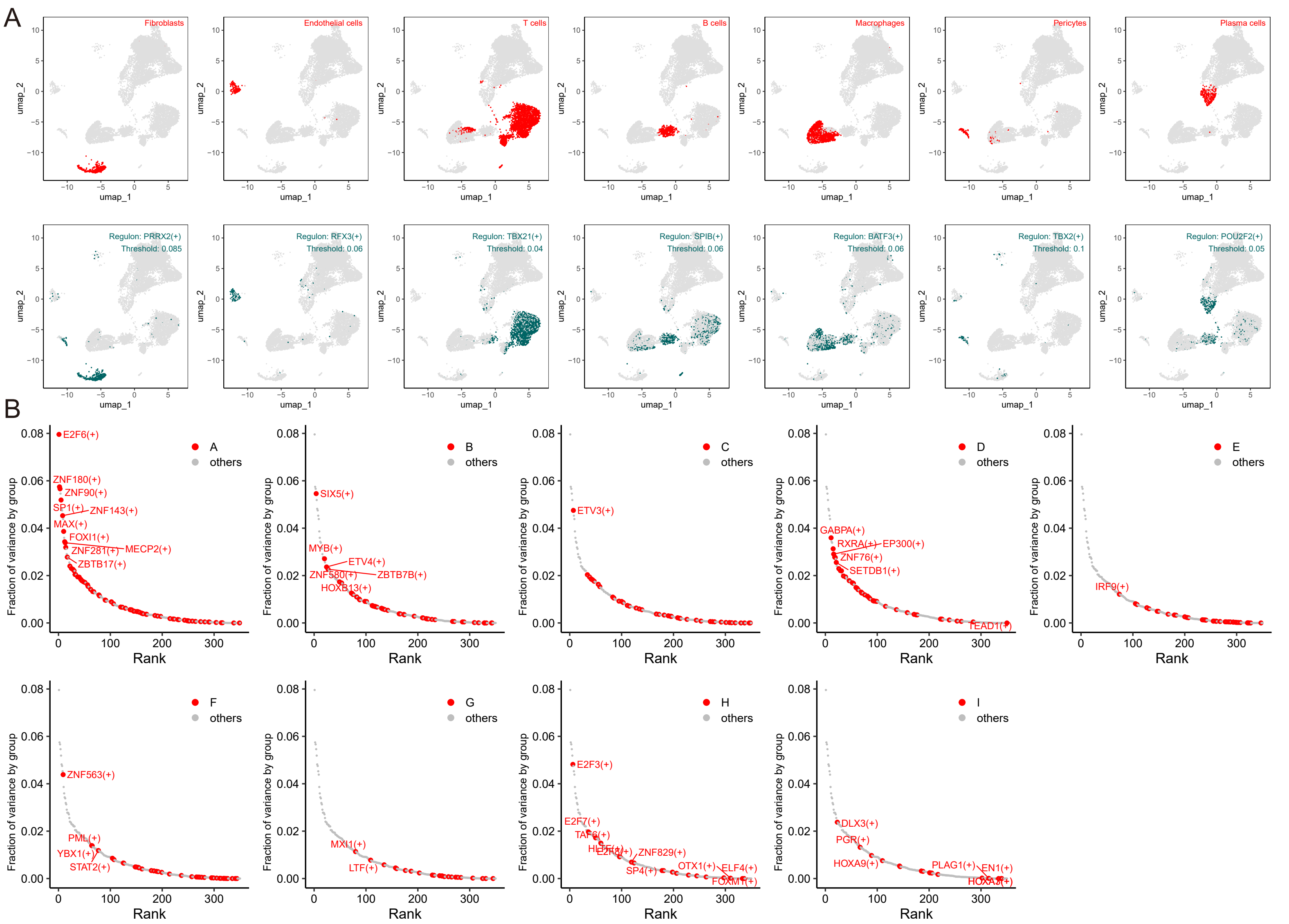

Figure S4. Detailed Transcription Factor Regulatory Analysis. (A) UMAP plot categorizing samples by transcription factor activity levels, visually illustrating groupings based on regulatory activity. (B) Contributions of transcription factor groups to AIRS, ranked by their Regulon Specificity Score (RSS); key transcription factors with significant contributions are highlighted.
