# Supplementary material for: Artificial intelligence-assisted RNA-binding protein signature for prognostic stratification and therapeutic guidance in breast cancer
Source: Front Immunol. 2025 Apr 16;16:1583103. doi: 10.3389/fimmu.2025.1583103 (PMC12040944; doi:10.3389/fimmu.2025.1583103)
Supplement: Supplementary file 1 [file DataSheet1.pdf]

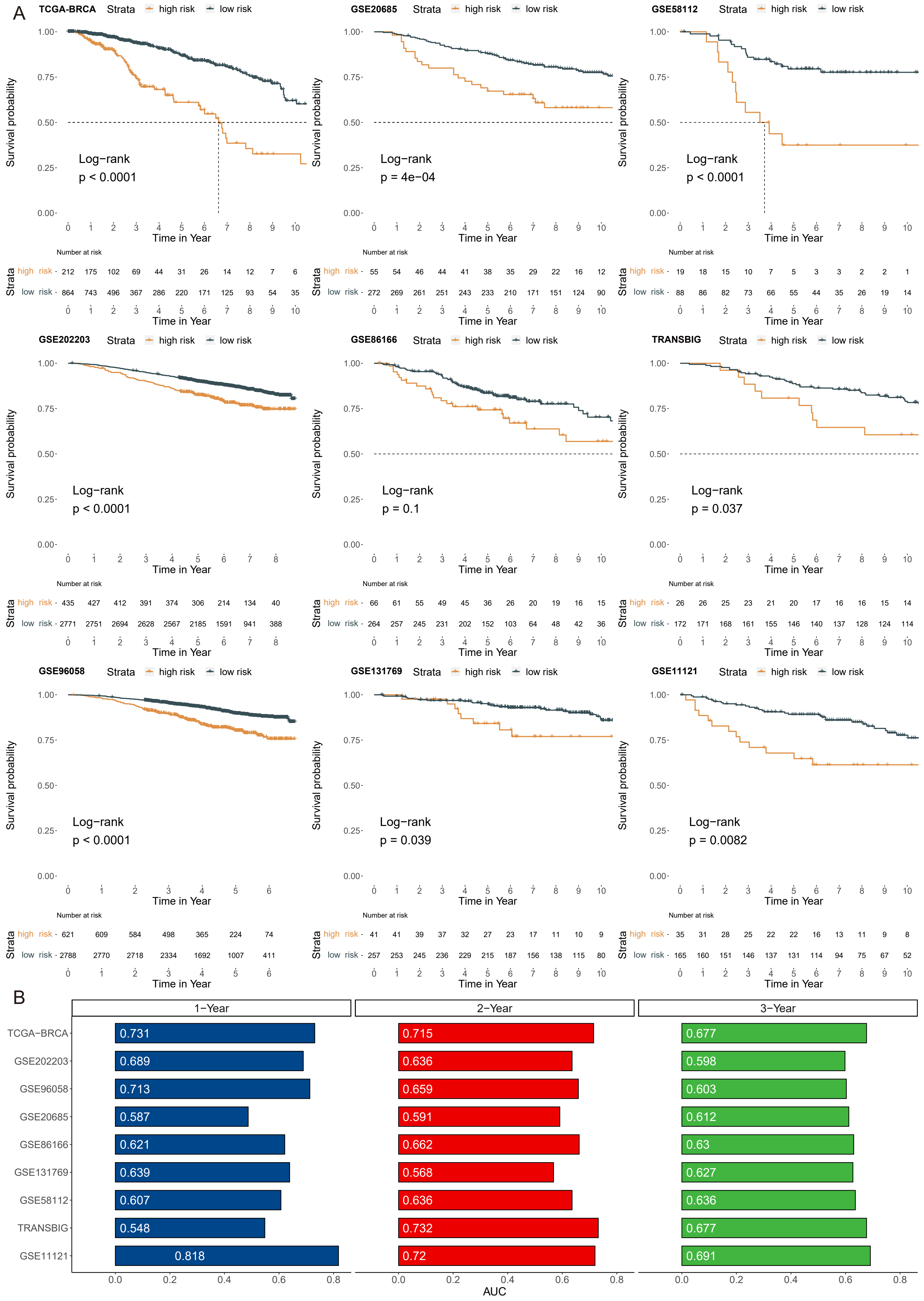

Figure S1. Kaplan-Meier survival analysis of the AIRS model. (A) Kaplan-Meier survival curves comparing high-risk and low-risk groups across nine cohorts, demonstrating significant differences in survival outcomes. (B) Time-dependent area under the curve (AUC) values for the AIRS model in nine training cohorts, illustrating its strong predictive capability for 1-year, 3-year, and 5-year survival probabilities.
